# Cancer-Associated Pulmonary Embolism in Contemporary Clinical Practice: Clinical Severity, Incidental Detection, and Early Outcomes in a Real-World Cohort

**DOI:** 10.3390/jcm15156092

**Published:** 2026-08-05

**Authors:** Călin Pop, Viorel Manea, Lucian Liviu Pop, Roxana Hodas, Lavinia Pop, Raluca Stefana Ioana Moș, Iulia Pop

**Affiliations:** 1 2nd Department, Faculty of Nursing and Health Sciences, “Iuliu Hatieganu” University of Medicine—Cluj Napoca, Str. Louis Pasteur Nr. 4, 400349 Cluj Napoca, Romania; 2 Department of Cardiology, “Constantin Opriş” Emergency County Hospital, Str. George Cosbuc Nr. 31, 430130 Baia Mare, Romania; 3 Department of Medicine, “Vasile Goldiș” Western University, B-dul Revoluției Nr. 96, 310025 Arad, Romania

**Keywords:** pulmonary embolism, cancer-associated thrombosis, incidental pulmonary embolism, right ventricular dysfunction, risk stratification, PESI, sPESI, venous thromboembolism

## Abstract

**Background**: The increasing use of routine oncological imaging has led to more frequent detection of incidental pulmonary embolism (PE), potentially modifying the contemporary clinical presentation of cancer-associated pulmonary embolism (CAPE). **Methods**: We performed a retrospective cohort study including 381 consecutive patients hospitalized with acute PE, of whom 58 had active cancer and 323 had no active malignancy. The primary endpoint was a Severe Hemodynamic Presentation Composite Endpoint (SHPCE), defined as shock, systolic blood pressure < 90 mmHg, and/or high-risk PE according to European Society of Cardiology criteria. Clinical characteristics, severity markers, management strategies, and in-hospital outcomes were compared between patients with CAPE and non-cancer PE (NCPE). Multivariable logistic regression analyses were performed to evaluate factors associated with SHPCE and incidental PE. **Results**: The patients with CAPE had higher PESI (129.8 ± 29.3 vs. 110.1 ± 32.3; *p* < 0.001) and sPESI scores (3.03 ± 0.56 vs. 2.68 ± 0.81; *p* < 0.001), and lower hemoglobin levels (11.7 ± 1.9 vs. 13.2 ± 1.8 g/dL; *p* < 0.001). Incidental PE was more frequent in CAPE than NCPE cases (13.8% vs. 1.9%; OR 8.45, 95% CI 2.81–25.39; *p* < 0.001). Despite their higher baseline risk scores, patients with CAPE and NCPE showed similar rates of SHPCE (12.1% vs. 17.6%; *p* = 0.295), ICU admission (10.3% vs. 11.5%; *p* = 1.000), and in-hospital mortality (10.3% vs. 11.1%; *p* = 0.858). In multivariable analyses, right ventricular dysfunction (RVD) showed the strongest association with SHPCE (adjusted OR 10.21, 95% CI 5.34–19.52; *p* < 0.001), whereas active cancer was not associated with severe presentation. **Conclusions**: Active cancer was associated with higher clinical risk scores and a greater prevalence of incidental PE but not with increased hemodynamic severity or adverse in-hospital outcomes. Acute PE severity appeared to be more closely related to right ventricular involvement than to cancer status, supporting a severity-based rather than cancer-based approach to risk assessment.

## 1. Introduction

Pulmonary embolism (PE), the third leading cause of cardiovascular death worldwide after myocardial infarction and stroke, continues to represent a major public health burden despite substantial advances in diagnostic imaging, risk stratification, and treatment [1,2,3,4,5]. According to contemporary European Society of Cardiology (ESC) and American Heart Association (AHA) reports, PE accounts for more than 300,000 deaths annually in Europe and remains associated with in-hospital mortality rates ranging from 5% to 15% depending on disease severity and baseline patient characteristics [2,3,4,5]. Contemporary management increasingly emphasizes early risk stratification based on hemodynamic status, right ventricular dysfunction (RVD), and biomarkers.

Active malignancy is one of the strongest acquired risk factors for venous thromboembolism (VTE). Patients with cancer have a four- to seven-fold higher risk of VTE than the general population, and approximately 15–20% of all VTE events occur in patients with active cancer [6,7,8]. Thromboembolic disease complicates the clinical course of up to 20% of cancer patients and remains the second leading cause of death after cancer progression [9,10].

The biological relationship between cancer and thrombosis is multifactorial and involves tissue factor expression, platelet activation, endothelial dysfunction, inflammatory pathways, and extracellular vesicle release [11,12,13,14]. Consequently, cancer-associated thrombosis (CAT) has traditionally been regarded as a severe clinical condition associated with recurrent VTE, bleeding complications, and mortality [15,16,17,18].

Large registries and randomized trials have consistently demonstrated the adverse prognosis associated with CAT. In the RIETE registry, active cancer was associated with higher rates of recurrent VTE and death during follow-up [19]. Similarly, the Hokusai-VTE Cancer, SELECT-D, ADAM VTE, and Caravaggio studies confirmed the substantial burden of cancer-associated thromboembolic disease despite advances in anticoagulant therapy [20,21,22,23]. However, these studies primarily focused on recurrent VTE, bleeding complications, and long-term survival rather than on the severity of the initial PE presentation.

Contemporary oncology practice has profoundly modified the diagnostic landscape of PE. The widespread use of contrast-enhanced pulmonary computed tomography (CT) for staging and surveillance has increased the detection of incidental PE. Recent studies indicate that incidental PE accounts for approximately 10–30% of PE diagnoses in cancer patients [24,25,26,27]. Several investigations have also suggested that patients with incidental PE present with fewer signs of hemodynamic compromise and may experience a more favorable short-term clinical course than those with symptomatic PE [24,25,26,27]. Consequently, contemporary CAPE cohorts may include a substantial proportion of patients diagnosed before the development of overt hemodynamic compromise.

These observations raise an important question. While active malignancy clearly worsens long-term prognosis, it remains uncertain whether CAPE necessarily presents as a more severe acute thromboembolic syndrome at hospital admission. Increasing evidence suggests that early outcomes are driven primarily by hemodynamic instability, right ventricular dysfunction (RVD), myocardial injury, and clot burden rather than by cancer status itself [3,4,5,28,29,30].

We hypothesized that, although active cancer substantially increases the risk of VTE, the contemporary clinical presentation of patients with CAPE is influenced by the increasing detection of incidental PE and is therefore not necessarily associated with greater acute hemodynamic severity or worse early in-hospital outcomes than in patients with non-cancer pulmonary embolism (NCPE). We further hypothesized that acute PE severity would be more closely related to hemodynamic impairment and RVD than to cancer status itself.

Therefore, the primary objective of the present study was to compare the clinical presentation, severity profile, management strategies, and in-hospital outcomes of patients with CAPE and NCPE in a consecutive real-world cohort.

The secondary objectives were to identify factors associated with severe hemodynamic presentation and incidental PE detection among cancer patients.

## 2. Materials and Methods

### 2.1. Study Design and Population

This retrospective, single-center observational cohort study was conducted in the Department of Cardiology and Cardiovascular Intensive Care at the “Dr. Constantin Opriș” Emergency County Hospital, Baia Mare, Romania, a regional secondary/tertiary referral center. Consecutive adult patients hospitalized with definitive PE between 1 January 2023, and 31 December 2025, were included. The hospital’s Institutional Review Ethics Committee approved the study protocol on 2 February 2026 (decision no. 4983). Due to the retrospective design and use of anonymized routinely collected clinical data, the requirement for individual informed consent was waived in accordance with institutional regulations. The study followed the STROBE recommendations for observational studies [31].

Final diagnoses of PE were established using computed tomography pulmonary angiography (CTPA) according to contemporary ESC recommendations [2]. Patients younger than 18 years and those without objective imaging confirmation of PE were excluded from the study. For the present analysis, patients were classified into two groups according to the presence or absence of active malignancy:

- Cancer-associated pulmonary embolism (CAPE).
- Non-cancer pulmonary embolism (NCPE).

Because of the retrospective observational design, no a priori sample size calculation was performed. The study included all consecutive eligible patients diagnosed during the predefined study period, providing a real-world cohort for exploratory analyses.

### 2.2. Definitions

#### 2.2.1. Malignancy 

Active cancer was defined according to the International Society on Thrombosis and Haemostasis (ISTH) criteria and previous cancer-associated thrombosis studies as any of the following: newly diagnosed malignancy within the previous 6 months; recurrent or metastatic disease; ongoing anticancer treatment (chemotherapy, immunotherapy, targeted therapy, hormonal therapy, or radiotherapy) [7,8,10,15]. Patients with a distant past of malignancy without evidence of active disease were classified as non-cancer patients. Active anticancer treatment was coded as present only in patients receiving ongoing cancer-directed therapy; all other patients were classified as not receiving active treatment.

#### 2.2.2. PE Types

Incidental PE was defined as PE identified on contrast-enhanced CT imaging performed for reasons unrelated to suspected acute PE, including cancer staging, treatment monitoring, or routine oncological surveillance [24,25,26]. Cases without documentation of incidental diagnosis were classified as symptomatic PE.

#### 2.2.3. Hemodynamic Characteristics

Hemodynamic instability was defined according to ESC 2024 and AHA scientific statement definitions as cardiac arrest, obstructive shock, or persistent hypotension. Obstructive shock was defined as a systolic blood pressure (SBP) < 90 mmHg or the need for vasopressor support to maintain SBP ≥ 90 mmHg, accompanied by signs of end-organ hypoperfusion. Persistent hypotension was defined as SBP < 90 mmHg or a decrease in SBP ≥ 40 mmHg from baseline lasting longer than 15 min in the absence of an alternative cause [2,3].

#### 2.2.4. RVD

RVD was considered present when imaging demonstrated right ventricular (RV) dilatation, systolic dysfunction, pressure overload, or an increased RV/left ventricular (LV) diameter ratio on echocardiography and/or CTPA [2,3,4].

#### 2.2.5. Deep Vein Thrombosis

Deep vein thrombosis (DVT) was defined as objectively confirmed lower-extremity DVT diagnosed by compression ultrasonography [2,3].

### 2.3. Data Collection

Clinical, laboratory, imaging, treatment, and outcome data were extracted from electronic medical records.

#### 2.3.1. Baseline Variables

The following baseline characteristics were recorded: age; sex; smoking status; obesity (body mass index—BMI > 30, according to World Health Organisation criteria); previous VTE; recent surgery; recent trauma; immobilization; known thrombophilia; pregnancy/postpartum status [32].

#### 2.3.2. Comorbidities 

Comorbidities included the following: arterial hypertension (HTN); diabetes mellitus (DM); coronary artery disease (CAD); chronic heart failure (CHF); atrial fibrillation (AF); a history of chronic kidney disease (CKD) (which was recorded according to the diagnostic criteria documented in the medical records and classified in accordance with KDIGO 2024 recommendations); chronic obstructive pulmonary disease (COPD); previous stroke [33].

#### 2.3.3. Clinical Presentation

Symptoms recorded at admission included dyspnea; chest pain; hemoptysis; syncope; lower-limb pain; lower-limb edema. Vital signs included SBP; heart rate (HR); oxygen saturation (SaO_2_).

#### 2.3.4. Laboratory Variables

Laboratory assessment included D-dimer; cardiac troponin; NT-proBNP; hemoglobin; platelet count; serum creatinine. Myocardial injury was defined as troponin concentrations above the upper reference limit of the local laboratory assay [2,3,4].

#### 2.3.5. Imaging Variables

CTPA variables: central PE; lobar PE; segmental/subsegmental PE; bilateral PE.

Echocardiographic variables: RV dilatation; pulmonary artery systolic pressure (sPAP); tricuspid annular plane systolic excursion (TAPSE). RV/LV diameter ratio was included when recorded. Presence of concomitant DVT was also recorded.

### 2.4. Risk Stratification

PE severity was assessed using the following indices:

- Pulmonary Embolism Severity Index (PESI): the original PESI score was calculated according to the model proposed by Aujesky et al. [34].
- Simplified PESI (sPESI): the simplified PESI score was calculated according to the model proposed by Jiménez et al. [35].
- ESC risk classification: patients were categorized as low risk; intermediate–low risk; intermediate–high risk; or high-risk PE, according to ESC guidelines integrating hemodynamic status, RVD, and cardiac biomarkers [2].

### 2.5. Treatment Variables

The following therapeutic variables were analyzed: anticoagulant strategy; systemic thrombolysis; inferior vena cava filter implantation; and intensive care unit (ICU) admission.

### 2.6. Study Objectives and Outcomes

The primary objective of this study was to compare the clinical presentation, severity profile, management strategies, and in-hospital outcomes of patients with CAPE or NCPE.

The secondary objectives were (i) to identify clinical and imaging factors associated with severe hemodynamic presentation of PE, and (ii) to explore factors associated with incidental PE detection among patients with active cancer.

#### 2.6.1. Primary Endpoint

The primary endpoint was the Severe Hemodynamic Presentation Composite Endpoint (SHPCE), defined as the occurrence of at least one of the following at presentation or during the early hospital course: shock, SBP < 90 mmHg, or high-risk PE according to ESC risk stratification criteria [2].

#### 2.6.2. Secondary Endpoints and Outcomes

Secondary outcomes were grouped into three predefined domains:

- Early clinical outcomes: in-hospital mortality; length of hospital stay; recurrent VTE; major bleeding.
- Management-related outcomes: ICU admission; systemic thrombolysis; inferior vena cava filter implantation.
- PE presentation and severity markers: incidental PE; PESI and sPESI scores; RVD; elevated troponin; PE localization; concomitant DVT.

Major bleeding was defined according to ISTH criteria [36].

Two investigators independently identified cases and verified data. A senior cardiologist resolved discrepancies through adjudication.

## 3. Statistical Analysis

Continuous variables were tested for normality using the Shapiro–Wilk test and are presented as mean ± standard deviation (SD) or median (interquartile range, IQR), as appropriate. Categorical variables are presented as counts and percentages. Comparisons between patients with CAPE and NCPE were performed using Student’s *t*-test or the Mann–Whitney U test for continuous variables and the χ^2^ test or Fisher’s exact test for categorical variables.

The primary analysis compared baseline characteristics, PE severity markers, management strategies, and in-hospital outcomes between CAPE and NCPE patients.

To address the secondary study objectives, two exploratory logistic regression models were constructed: (i) a model evaluating factors associated with the SHPCE in the overall cohort and (ii) a model evaluating factors associated with incidental PE among cancer patients. Variables were selected according to clinical relevance, biological plausibility, and univariable associations for *p* value < 0.10. Variables included in the definition of SHPCE, as well as the PESI and sPESI scores, were not entered into multivariable models to avoid collinearity [37].

As a prespecified sensitivity analysis, all comparisons were repeated after exclusion of incidentally diagnosed PE cases. Results are reported as odds ratios (ORs) with 95% confidence intervals (CIs). Multivariable analyses were considered exploratory and hypothesis-generating. All tests were two-sided, and *p* < 0.05 was considered statistically significant. Statistical analyses were performed using IBM SPSS Statistics, Version 29.0 (IBM Corp., Armonk, NY, USA).

## 4. Results

### 4.1. Baseline Characteristics

A total of 381 patients with acute PE were included, of whom 58 (15.2%) had CAPE and 323 (84.8%) had NCPE (Table 1).

The baseline demographic characteristics, comorbidities, presenting symptoms, vital signs, and most laboratory and imaging findings were comparable between the groups. Recent surgery was more frequent among the patients with CAPE (24.1% vs. 12.4%; *p* = 0.024), whereas AF was less prevalent (13.8% vs. 29.1%; *p* = 0.015). The patients with CAPE also had lower hemoglobin levels (11.7 ± 1.9 vs. 13.2 ± 1.8 g/dL; *p* < 0.001).

No significant differences were observed in the biomarkers of PE severity, including troponin and NT-proBNP, or in imaging indicators such as PE localization, RVD, RV/LV ratio, TAPSE, sPAP, and concomitant DVT. A trend toward a lower prevalence of central PE was observed among cancer patients (1.7% vs. 5.6%; *p* = 0.08).

Incidental PE was substantially more frequent in patients with CAPE, than in those with NCPE (13.8% vs. 1.9%; *p* < 0.001). Despite similar biological and imaging markers of PE severity, the patients with CAPE exhibited significantly higher PESI (129.8 ± 29.3 vs. 110.1 ± 32.3; *p* < 0.001) and sPESI scores (3.03 ± 0.56 vs. 2.68 ± 0.81; *p* < 0.001).

### 4.2. Cancer Characteristics and Mode of PE Diagnosis

The patients’ cancer-related characteristics are summarized in Table 2. Gastrointestinal (34.5%) and thoracic malignancies (24.1%) were the most common cancer types, and 89.7% of the patients with CAPE were receiving active anticancer treatment.

Incidental PE accounted for 13.8% of the CAPE cases and was observed predominantly in patients undergoing active oncological treatment. No patient with incidental PE experienced SHPCE, shock, high-risk PE, ICU admission, thrombolytic therapy, or in-hospital death. In contrast, all severe clinical events that were recorded occurred among the symptomatic patients with CAPE. Given the limited number of incidental events (n = 8), these findings should be considered descriptive.

### 4.3. Clinical Presentation Severity and In-Hospital Outcomes

The clinical presentation severity and in-hospital outcomes are summarized in Table 3.

The primary endpoint, SHPCE, occurred in 12.1% of the patients with CAPE and 17.6% of the patients with NCPE (*p* = 0.295). Similarly, shock (6.9% vs. 13.0%), high-risk PE (5.2% vs. 8.7%), ICU admission (10.3% vs. 11.5%), thrombolysis (1.7% vs. 5.3%), and in-hospital mortality (10.3% vs. 11.1%) did not differ significantly between the groups.

Initial anticoagulant treatment after PE diagnosis was similar between groups, with low-molecular-weight heparin (LMWH) being the most frequently used therapy in both patients with CAPE (69.0%) and NCPE (74.3%), whereas unfractionated heparin was administered mainly to patients presenting with greater clinical instability.

A supplementary subgroup analysis of patients requiring ICU admission showed that ICU care was required in 6/58 (10.3%) patients with CAPE and 37/323 (11.5%) with NCPE. Within this subgroup, shock was present in 3/6 (50.0%) patients with CAPE and 26/37 (70.3%) patients with NCPE, while RVD was documented in 5/6 (83.3%) and 28/37 (75.7%), respectively. In-hospital mortality remained high in both groups, occurring in 5/6 (83.3%) patients with CAPE and 29/37 (78.4%) patients with NCPE. Systemic thrombolysis was administered to only 1/6 (16.7%) ICU patients with CAPE and 4/37 (10.8%) ICU patients with NCPE.

Overall, despite higher baseline clinical risk scores, the patients with CAPE did not exhibit greater acute PE severity, increased escalation of care, or worse in-hospital outcomes.

### 4.4. Factors Associated with Severe Hemodynamic Presentation

Exploratory logistic regression analyses were performed to identify factors associated with SHPCE (Table 4).

In the univariable analyses, RVD, CKD, and increasing age were associated with severe presentation. In the multivariable model, RVD showed the strongest association with SHPCE (adjusted OR 10.21, 95% CI 5.34–19.52; *p* < 0.001), whereas active cancer, active anticancer treatment, age, CKD, concomitant DVT, and positive troponin were not significantly associated with severe presentation after adjustment. Incidental PE showed an inverse association with SHPCE but did not reach statistical significance.

Overall, markers of RV involvement appeared more closely related to acute PE severity than cancer status itself.

### 4.5. Factors Associated with Incidentally Diagnosed Pulmonary Embolism

An exploratory regression analysis was performed to evaluate factors associated with incidental PE among cancer patients (Table 5).

Active anticancer treatment (adjusted OR 6.41, 95% CI 0.72–57.4) and thoracic malignancies (adjusted OR 3.52, 95% CI 0.72–17.1) showed the strongest associations with incidental PE detection, whereas RVD showed an inverse association (adjusted OR 0.41, 95% CI 0.05–3.84). Although none of these associations reached statistical significance, their direction was consistent with the descriptive findings observed in Table 2. Given the limited number of incidental events, these results should be regarded as hypothesis-generating.

### 4.6. Sensitivity Analysis Excluding Incidental Pulmonary Embolism

To assess the influence of incidental PE, all comparisons were repeated after exclusion of incidentally diagnosed cases (Table 6).

The patients with symptomatic CAPE continued to exhibit higher PESI (131.8 ± 28.7 vs. 110.1 ± 32.3; *p* < 0.001) and sPESI scores (3.05 ± 0.55 vs. 2.68 ± 0.81; *p* < 0.001), as well as lower hemoglobin levels (11.6 ± 1.9 vs. 13.2 ± 1.8 g/dL; *p* < 0.001), than the symptomatic patients with NCPE.

In contrast, the SHPCE rates remained comparable (14.0% vs. 18.0%; *p* = 0.492), and no meaningful differences were observed regarding biological markers, imaging findings, management strategies, or early clinical outcomes (Supplementary Table S1).

Overall, the sensitivity analysis yielded findings consistent with the primary analysis, indicating that the similarities in acute PE severity and short-term outcomes between CAPE and NCPE persisted after the exclusion of incidental PE cases.

## 5. Discussion

The main finding of the present study is that active cancer was associated with higher baseline clinical risk scores but not with a more severe acute presentation of PE. Although the patients with CAPE exhibited significantly higher PESI and sPESI scores, the rates of severe hemodynamic presentation, escalation of care, and in-hospital mortality were like those observed in patients. Furthermore, exploratory multivariable analyses showed that markers of RV involvement were more closely associated with severe presentation than cancer status itself. Collectively, these findings suggest that the contemporary clinical phenotype of patients with CAPE may differ from the traditional perception of CAT as an inherently more severe thromboembolic condition.

These observations should be interpreted within the context of the evolving epidemiology of CAT. Active cancer remains one of the strongest acquired risk factors for VTE and is consistently associated with increased long-term mortality, recurrent VTE, and bleeding complications. Large registries and randomized trials, including RIETE, Hokusai-VTE Cancer, SELECT-D, ADAM-VTE, and Caravaggio, have established the adverse long-term prognosis associated with CAT. However, these studies primarily focused on recurrent thromboembolic events, bleeding complications, and survival rather than on the severity of the initial PE presentation. Consequently, whether cancer patients present with a more severe acute PE syndrome remains less clearly defined [19,20,21,22,23].

A noteworthy observation in our cohort was the apparent discrepancy between baseline risk stratification and acute clinical severity. Patients with CAPE had significantly higher PESI and sPESI scores, yet they did not experience higher rates of SHPCE, shock, hypotension, high-risk PE, ICU admission, thrombolysis, or in-hospital mortality. This finding is biologically plausible because cancer status contributes directly to both PESI and sPESI calculations. Consequently, these scores may appropriately reflect overall prognosis while being less specific for estimating the risk of acute hemodynamic deterioration in oncology patients. Similar observations have been reported in contemporary studies, suggesting that prognostic scores incorporating cancer status may be more predictive of medium-term mortality than of immediate hemodynamic compromise [38,39,40].

Another important observation was the markedly higher prevalence of incidental PE among cancer patients. Incidental PE was approximately eight times more frequent in patients with CAPE than in NCPE cases and was not accompanied by severe hemodynamic events, ICU admission, thrombolysis, or in-hospital death. These findings are consistent with previous reports showing that incidental PE accounts for approximately 10–30% of PE diagnoses in oncology populations owing to the widespread use of contrast-enhanced CT imaging for cancer staging and surveillance. Several studies have further demonstrated that incidentally detected PE is frequently associated with lower clot burden, fewer signs of cardiopulmonary compromise, and more favorable short-term outcomes [24,25,26,27,41,42,43].

The exploratory analysis evaluating factors associated with incidental PE provides additional context for these observations. Active anticancer treatment and thoracic malignancies showed the strongest associations with incidental PE detection, whereas RVD showed an inverse association. Although these associations did not reach statistical significance and should be interpreted cautiously, their direction is consistent with the descriptive findings observed in our cohort. Together, these observations support the hypothesis that the higher prevalence of incidental PE among cancer patients is largely related to repeated oncological imaging and earlier event detection rather than to intrinsic differences in thrombus biology. The predominance of thoracic malignancies among incidental cases further supports this interpretation, as these patients routinely undergo serial chest CT examinations during treatment and follow-up.

Perhaps the most clinically relevant finding of the present study was that RVD showed the strongest association with severe hemodynamic presentation, whereas active cancer was not associated with SHPCE after adjustment for clinically relevant covariates. These findings are consistent with contemporary ESC and AHA recommendations, which identify hemodynamic instability and RVD as the principal determinants of early PE prognosis. This observation is biologically plausible, as acute clinical deterioration is primarily driven by the extent of pulmonary vascular obstruction and the resulting RV pressure overload, rather than by the underlying mechanism predisposing to thrombosis. Thus, although active cancer substantially increases the risk of VTE, it does not necessarily aggravate the acute cardiopulmonary consequences once PE has occurred. Accordingly, acute risk stratification and management should continue to rely on objective markers of hemodynamic compromise and RVD rather than on the presence of active malignancy alone. This concept was further supported by the ICU subgroup analysis, in which patients with CAPE and NCPE showed similarly high rates of shock, RVD, and in-hospital mortality despite contemporary management. Although systemic thrombolysis was administered to a small number of critically ill patients, particularly in the CAPE group, the limited sample precludes meaningful conclusions regarding its efficacy according to cancer status. Overall, our findings, together with evidence from large registries and contemporary risk stratification studies, indicate that the severity of acute PE is determined predominantly by acute cardiopulmonary impairment rather than by cancer status itself [2,3,4,5,44,45,46,47].

The sensitivity analysis restricted to symptomatic PE further supports this interpretation. After exclusion of incidental events, patients with CAPE continued to exhibit higher PESI and sPESI scores and lower hemoglobin levels, whereas biological markers, imaging findings, clinical severity, management requirements, and early outcomes remained broadly comparable between groups. Notably, no significant differences were observed regarding SHPCE, shock, high-risk PE, ICU admission, thrombolysis, recurrent VTE, major bleeding, or in-hospital mortality.

Similarly, biomarkers of PE severity, including troponin and NT-proBNP, as well as imaging indicators such as RVD, RV/LV ratio, TAPSE, sPAP, and concomitant DVT, remained comparable. Taken together, these findings suggest that the similarities observed between patients with CAPE and NCPE, are not solely explained by the inclusion of incidental PE cases and persist even when analyses are restricted to symptomatic presentations.

Initial anticoagulant management was broadly comparable between the CAPE and NCPE groups, with LMWH representing the predominant initial in-hospital treatment, consistent with contemporary guideline recommendations for the management of acute PE. Current ESC, AHA, American Society of Hematology, and American Society of Clinical Oncology guidelines recommend long-term anticoagulation with a DOAC whenever appropriate, while recognizing that LMWH remains an appropriate alternative for selected patients with active cancer, particularly those at increased bleeding risk or with gastrointestinal or genitourinary malignancies. However, information on anticoagulant therapy before hospital admission (including prophylactic or therapeutic dosing and treatment indication) and at hospital discharge was not systematically collected in our retrospective database. Consequently, the potential influence of pre-existing or discharge anticoagulation strategies on clinical presentation, acute severity, and outcomes of PE could not be formally evaluated [2,3,12,48].

The lower hemoglobin levels observed among cancer patients deserve specific consideration. Anemia is highly prevalent in oncology populations and reflects a complex interplay between chronic inflammation, nutritional deficiencies, bone marrow suppression, and treatment-related toxicity. Although hemoglobin levels were significantly lower in the patients with CAPE, anemia was not associated with severe hemodynamic presentation or adverse short-term outcomes. This finding suggests that lower hemoglobin concentrations may contribute to higher prognostic scores without necessarily translating into greater acute PE severity. Similar observations have been reported in studies evaluating prognostic markers in cancer-associated thrombosis [49,50].

Another interesting observation was the lower prevalence of AF among patients with CAPE. Although AF is frequently considered a marker of cardiovascular comorbidity and frailty in PE populations, its lower frequency in patients with CAPE likely reflects differences in baseline patient characteristics, rather than a direct relationship with thromboembolic presentation. Nevertheless, this finding further illustrates that CAPE and NCPE patients may differ in several clinical dimensions despite exhibiting similar acute outcomes.

Finally, the heterogeneity of the cancer population in our study may also partly explain the absence of a more severe acute presentation in the CAPE group. Different tumor types, disease stages, and anticancer therapies are associated with variable prothrombotic potential and different intensities of imaging surveillance, influencing both the biological risk of thrombosis and the likelihood of detecting incidental PE. Accordingly, the contemporary clinical phenotype of patients with CAPE probably reflects the combined effects of cancer biology and evolving oncological practice rather than cancer status alone.

### 5.1. Clinical Implications

An important clinical implication of our findings is the distinction between acute PE severity and long-term prognosis in patients with cancer. Although active malignancy is a well-established determinant of recurrent VTE, bleeding complications, and long-term mortality, it was not associated with a more severe hemodynamic presentation at the time of acute PE in our cohort. These findings suggest that cancer status alone should not be interpreted as a marker of greater acute PE severity. Instead, assessment of the initial clinical risk should continue to rely on established indicators of hemodynamic compromise and careful assessment of RVD, while the presence of active cancer remains a key determinant of long-term management and prognosis.

Second, the increasing prevalence of incidental PE highlights the importance of systematically evaluating surveillance CT scans in oncology practice and supports current recommendations advocating similar anticoagulation strategies for incidental and symptomatic PE when clinically appropriate.

Finally, prognostic scores incorporating cancer status should be interpreted cautiously when estimating the risk of acute hemodynamic deterioration [2,4,24,40].

### 5.2. Study Limitations

Several limitations should be acknowledged. Although consecutive patients were included, the retrospective single-center design may be subject to selection bias and residual confounding. Despite adjustment for clinically relevant variables, unmeasured factors —including cancer stage, performance status, treatment-related characteristics, and referral patterns—may have influenced thrombotic risk, mode of PE presentation, and clinical outcomes. Therefore, multivariable analyses should be regarded as exploratory and hypothesis-generating rather than definitive prognostic models.

Second, the number of cancer patients, particularly those with incidental PE, was relatively small, limiting statistical power and the precision of multivariable estimates.

Third, the heterogeneity of malignancy types and oncological treatments may have influenced clinical presentation and outcomes. Information on the specific type of anticancer therapy was not systematically available. Consequently, although most patients with CAPE (89.7%) were receiving active oncological treatment, the potential influence of different treatment modalities on PE severity and short-term outcomes could not be evaluated.

Fourth, information regarding anticoagulant therapy before hospital admission (including prophylactic or therapeutic dosing and treatment indication), discharge anticoagulant regimen, and long-term treatment adherence was not systematically collected. Therefore, the potential influence of different anticoagulation strategies on the clinical presentation of PE and subsequent outcomes could not be evaluated.

Finally, only in-hospital outcomes were evaluated, precluding conclusions regarding long-term prognosis.

### 5.3. Future Directions

Several questions raised here remain unanswered and warrant further investigation. Larger multicenter studies are needed to confirm whether the lower apparent severity of patients with CAPE is primarily explained by the increasing prevalence of incidentally detected events.

Future analyses should also evaluate whether contemporary prognostic scores such as the PESI and sPESI adequately reflect the acute hemodynamic risk in oncology patients or whether cancer status disproportionately influences risk classification.

In addition, the prognostic implications of incidental PE according to cancer type, disease stage, and treatment modality remain incompletely understood.

Finally, prospective studies integrating imaging parameters, circulating biomarkers, and cancer-related variables may help identify distinct phenotypes of cancer-associated PE and improve individualized risk stratification.

## 6. Conclusions

In this study, active cancer was associated with higher prognostic risk scores and more frequent incidental PE but not with a more severe acute clinical presentation. The findings suggest that early PE severity is more closely related to cardiopulmonary involvement, particularly RVD, than to cancer status alone. The increasing detection of incidental PE through routine oncological imaging may partly influence the contemporary presentation of cancer-associated pulmonary embolism.

### Key Messages

- Patients with active cancer had higher PESI and sPESI scores but did not exhibit greater acute PE severity, higher rates of escalation of care, or worse in-hospital outcomes.
- Incidentally detected PE was substantially more frequent among cancer patients and was not accompanied by severe hemodynamic events or in-hospital mortality in this cohort.
- RVD showed the strongest association with severe hemodynamic presentation in exploratory multivariable analyses.
- Active anticancer treatment and thoracic malignancies were the factors most closely associated with incidental PE detection, supporting the role of routine oncological imaging in identifying clinically less severe events.
- Assessment of RV involvement remains central to acute PE risk stratification regardless of cancer status.
- Patients with CAPE represent a heterogeneous clinical entity that encompasses both incidentally detected low-severity events and clinically overt high-risk presentations.

## Figures and Tables

**Table 1:** Baseline characteristics of patients with cancer-associated pulmonary embolism (CAPE) and non-cancer pulmonary embolism (NCPE).

Variable	CAPE (n = 58)	NCPE (n = 323)	Effect Size (95% CI)	*p*-Value
Demographic Characteristics				
Age, years	71.1 ± 11.5	70.1 ± 13.3	-	0.553
Male sex, n (%)	28 (48.3)	173 (53.6)	-	0.478
Current smoker, n (%)	8 (13.8)	60 (18.6)	-	0.459
Obesity (BMI ≥ 30 kg/m^2^), n (%)	27 (46.6)	147 (45.5)	-	0.887
VTE Risk Factors				
Recent surgery, n (%)	14 (24.1)	40 (12.4)	OR 2.25 (1.14–4.43)	0.024
Recent trauma, n (%)	7 (12.1)	32 (9.9)	-	0.638
Immobilization, n (%)	16 (27.6)	91 (28.2)	-	1.000
Previous DVT, n (%)	3 (5.2)	44 (13.6)	OR 0.35 (0.11–1.14)	0.083
Previous PE, n (%)	1 (1.7)	30 (9.3)	OR 0.17 (0.02–1.24)	0.065
Known thrombophilia, n (%)	0 (0.0)	2 (0.6)	-	1.000
Pregnancy/postpartum, n (%) *	0 (0.0)	2 (0.6)	-	1.000
Comorbidities				
Hypertension, n (%)	34 (58.6)	196 (60.7)	-	0.772
Diabetes mellitus, n (%)	9 (15.5)	67 (20.7)	-	0.475
Coronary artery disease, n (%)	20 (34.5)	130 (40.2)	-	0.467
Chronic heart failure, n (%)	46 (79.3)	246 (76.2)	-	0.736
Atrial fibrillation, n (%)	8 (13.8)	94 (29.1)	OR 0.39 (0.18–0.84)	0.015
Chronic kidney disease, n (%)	10 (17.2)	41 (12.7)	-	0.401
COPD, n (%)	9 (15.5)	81 (25.1)	-	0.132
Previous stroke, n (%)	10 (17.2)	51 (15.8)	-	0.846
Clinical Presentation				
Dyspnea, n (%)	56 (96.6)	313 (96.9)	-	1.000
Chest pain, n (%)	37 (63.8)	214 (66.3)	-	0.764
Hemoptysis, n (%)	3 (5.2)	16 (5.0)	-	1.000
Syncope, n (%)	10 (17.2)	68 (21.1)	-	0.598
Lower-limb pain, n (%)	18 (31.0)	86 (26.6)	-	0.523
Lower-limb edema, n (%)	35 (60.3)	200 (61.9)	-	0.884
SBP, mmHg	127.1 ± 21.8	128.9 ± 26.4	-	0.630
Heart rate, bpm	99.3 ± 24.7	98.8 ± 22.8	-	0.890
Oxygen saturation, %	91.6 ± 5.9	92.1 ± 6.7	-	0.603
Shock, n (%)	4 (6.9)	42 (13.0)	-	0.272
Laboratory Findings				
Hemoglobin, g/dL	11.7 ± 1.9	13.2 ± 1.8	MD 1.47 (0.95 to 1.99)	<0.001
Creatinine, mg/dL	1.07 ± 0.8	1.00 ± 0.49	-	0.540
Platelet count, ×10^9^/L	207 (159–263)	196 (155–244)	-	0.765
D-dimer, ng/mL FEU	5.0 (5.0–6.0)	5.5 (5.0–6.5)	-	0.649
Troponin, ng/L	25.9 (11.1–122.5)	43.5 (11.3–158.5)	-	0.505
Elevated troponin (>14 ng/L), n/N (%)	31/47 (66.0)	191/266 (71.8)	-	0.486
NT-proBNP, pg/mL	1604 (270–5132)	2443 (475–7568)	-	0.226
Imaging Findings				
Bilateral PE, n (%)	37 (63.8)	214 (66.3)	-	0.764
Central PE, n (%)	1 (1.7%)	18 (5.6)	OR 0.30 (0.04–2.28)	0.08
Lobar PE, n (%)	43 (74.1)	211(65.3)	-	0.227
Segmental/subsegmental PE, n (%)	14 (24.1)	92 (28.5)	-	0.530
Right ventricular dysfunction, n (%)	15 (25.9)	91 (28.2)	-	0.874
RV/LV ratio	0.77 (0.65–0.93)	0.75 (0.65–0.86)	-	0.236
TAPSE, mm	18 (14–22)	19 (15–22)	-	0.971
sPAP, mmHg	40 (28–50)	37 (28–50)	-	0.781
Concomitant DVT, n (%)	15 (25.9)	85 (26.3)		1.000
Incidental PE, n (%)	8 (13.8)	6 (1.9)	OR 8.45 (2.81–25.39)	<0.001
Risk Stratification				
PESI score	129.8 ± 29.3	110.1 ± 32.3	MD 19.7 (11.1 to 28.3)	<0.001
sPESI score	3.03 ± 0.56	2.68 ± 0.81	MD 0.35 (0.15 to 0.55)	<0.001

Abbreviations: CAPE = cancer-associated pulmonary embolism; NCPE = non-cancer pulmonary embolism; BMI = body mass index; COPD = chronic obstructive pulmonary disease; DVT = deep vein thrombosis; SBP = systolic blood pressure; PE = pulmonary embolism; PESI = Pulmonary Embolism Severity Index; NT-proBNP = N terminal pro-B-type natriuretic peptide; sPAP = systolic pulmonary arterial pressure; sPESI = simplified Pulmonary Embolism Severity Index; VTE = venous thromboembolism. Statistically significant differences are highlighted by *p*-values < 0.05; * Ongoing pregnancy, postpartum < 6 weeks; data are presented as mean ± standard deviation (SD) or number (%), as appropriate; continuous variables are presented as mean ± SD or median (IQR), according to distribution; odds ratios (ORs) with 95% confidence intervals (CIs) are reported for categorical variables; mean differences (MDs) with 95% CIs are reported for continuous variables; for variables with missing data, percentages are calculated using available cases (e.g., troponin); elevated troponin was defined as >14 ng/L where troponin values were available.

**Table 2:** Cancer characteristics and mode of pulmonary embolism diagnosis in patients with cancer-associated pulmonary embolism (CAPE).

Characteristic	Overall CAPE (n = 58)	Incidental PE (n = 8)	Symptomatic PE (n = 50)
Cancer category			
Thoracic (lung), n (%)	14 (24.1%)	4 (50.0%)	10 (20.0%)
Gastrointestinal *, n (%)	20 (34.5%)	3 (37.5%)	17 (34.0%)
Breast/gynecologic **, n (%)	8 (13.8%)	0 (0.0%)	8 (16.0%)
Genitourinary ***, n (%)	13 (22.4%)	1 (12.5%)	12 (24.0%)
Hematological malignancies, n (%)	3 (5.2%)	0 (0.0%)	3 (6.0%)
Cancer status			
Active anticancer treatment, n (%)	52 (89.7%)	8 (100.0%)	44 (88.0%)
No active treatment, n (%)	6 (10.3%)	0 (0.0%)	6 (12.0%)
Mode of diagnosis			
Incidental PE, n (%)	8 (13.8%)	8 (100.0%)	—
Symptomatic PE, n (%)	50 (86.2%)	—	50 (100.0%)
Clinical severity and outcomes			
SHPCE, n (%)	7 (12.1%)	0 (0.0%)	7 (14.0%)
Shock, n (%)	4 (6.9%)	0 (0.0%)	4 (8.0%)
SBP < 90 mmHg, n (%)	4 (6.9%)	0 (0.0%)	4 (8.0%)
High-risk PE, n (%)	6 (10.3%)	0 (0.0%)	6 (12.0%)
ICU admission, n (%)	6 (10.3%)	0 (0.0%)	6 (12.0%)
Systemic thrombolysis, n (%)	1 (1.7%)	0 (0.0%)	1 (2.0%)
In-hospital mortality, n (%)	6 (10.3%)	0 (0.0%)	6 (12.0%)
Right ventricular dysfunction, n (%)	15 (25.9%)	0 (0.0%)	15 (30.0%)
Concomitant DVT, n (%)	15 (25.9%)	4 (50.0%)	11 (22.0%)

Abbreviations: CAPE, cancer-associated pulmonary embolism; DVT, deep vein thrombosis; ICU, intensive care unit; PE, pulmonary embolism; SBP, systolic blood pressure; SHPCE, Severe Hemodynamic Presentation Composite Endpoint. Data are presented as n (%); percentages in the overall CAPE column are calculated from 58 patients, while percentages in the incidental and symptomatic columns are calculated from 8 and 50 patients, respectively; comparisons between incidental and symptomatic PE are descriptive due to the small number of incidental cases. * Gastrointestinal cancers include colorectal, pancreatic, gastric, and hepatobiliary malignancies. ** Breast/Gynecologic cancers include breast and gynecologic malignancies. *** Genitourinary cancers include prostate, renal, and bladder/urothelial malignancies.

**Table 3:** Clinical presentation severity and in-hospital outcomes in patients with cancer-associated pulmonary embolism (CAPE) and non-cancer pulmonary embolism (NCPE).

Variable	CAPE (n = 58)	NCPE (n = 323)	*p*-Value
Primary Endpoint			
SHPCE *	7 (12.1%)	57 (17.6%)	0.295
Components of SHPCE			
Shock, n (%)	4 (6.9%)	42 (13.0%)	0.272
Systolic blood pressure < 90 mmHg, n (%)	4 (6.9%)	46 (14.2%)	0.176
High-risk pulmonary embolism, n (%)	6 (10.3%)	28 (8.7%)	0.589
Management-Related Outcomes			
Intensive care unit (ICU) admission, n (%)	6 (10.3%)	37 (11.5%)	1.000
Systemic thrombolysis, n (%)	1 (1.7%)	17 (5.3%)	0.331
Inferior vena cava filter implantation, n (%)	0 (0.0%)	5 (1.5%)	0.595
Low-molecular-weight heparin	40 (69%)	240 (74.3%)	0.396
Unfractionated heparin	18 (31%)	83 (25.7%)	0.396
Early Clinical Outcomes			
In-hospital mortality, n (%)	6 (10.3%)	36 (11.1%)	0.858
Recurrent VTE, n (%)	3 (5.2%)	37 (11.5%)	0.241
Major bleeding, n (%)	0 (0.0%)	4 (1.2%)	1.000
Length of hospital stay, days	8.9 ± 4.3	8.2 ± 3.8	0.214

Abbreviations: * Severe Hemodynamic Presentation Composite Endpoint (SHPCE): occurrence of at least one of the following during presentation or early hospitalization: shock, systolic blood pressure (SBP) < 90 mmHg, or high-risk pulmonary embolism according to ESC risk classification. The components of SHPCE were not mutually exclusive; therefore, individual event counts may overlap. CAPE, cancer-associated pulmonary embolism; ICU, intensive care unit; NCPE, non-cancer pulmonary embolism; SBP, systolic blood pressure; SHPCE, Severe Hemodynamic Presentation Composite Endpoint; VTE, venous thromboembolism.

**Table 4:** Predictors of Severe Hemodynamic Presentation Composite Endpoint (SHPCE): univariable and multivariable logistic regression analysis.

Variable	Univariable OR (95% CI)	*p*-Value	Multivariable Adjusted OR (95% CI)	*p*-Value
Active cancer	0.65 (0.28–1.52)	0.323	0.72 (0.29–1.81)	0.489
Active anticancer treatment	0.81 (0.39–1.70)	0.576	0.88 (0.38–2.05)	0.764
Incidental PE	0.14 (0.01–1.09)	0.061	0.18 (0.02–1.46)	0.108
Age (per year increase)	1.04 (1.01–1.06)	0.008	1.02 (0.99–1.05)	0.284
Male sex	1.38 (0.80–2.38)	0.243	1.29 (0.71–2.34)	0.398
Chronic kidney disease	2.45 (1.25–4.81)	0.009	1.73 (0.78–3.85)	0.177
Concomitant DVT	1.11 (0.59–2.10)	0.742	1.08 (0.54–2.14)	0.832
Positive troponin	1.98 (0.86–4.55)	0.109	1.41 (0.56–3.55)	0.462
Right ventricular dysfunction	11.64 (6.24–21.70)	<0.001	10.21 (5.34–19.52)	<0.001

Model performance: number of patients: 381; SHPCE events: 64; likelihood ratio test: *p* <0.001; Nagelkerke’s R^2^ = 0.23. Abbreviations: CI, confidence interval; DVT, deep vein thrombosis; OR, odds ratio; PE, pulmonary embolism; SHPCE, Severe Hemodynamic Presentation Composite Endpoint, defined as the occurrence of shock, SBP < 90 mmHg, and/or high-risk PE. Variables included in the multivariable model were selected according to clinical relevance, biological plausibility, and univariable associations. Odds ratios (ORs) are reported with 95% confidence intervals.

**Table 5:** Factors associated with incidentally diagnosed pulmonary embolism among patients with cancer-associated pulmonary embolism.

Variable *	Univariable OR (95% CI)	*p*-Value	Adjusted OR (95% CI)	*p*-Value
Active anticancer treatment	7.00 (0.81–60.8)	0.078	6.41 (0.72–57.4)	0.096
Thoracic malignancy	4.00 (0.87–18.4)	0.074	3.52 (0.72–17.1)	0.118
Right ventricular dysfunction	0.36 (0.04–3.16)	0.355	0.41 (0.05–3.84)	0.441
Concomitant DVT	3.55 (0.70–18.0)	0.125	2.84 (0.52–15.4)	0.225

Abbreviations: DVT, deep vein thrombosis; OR, odds ratio; CI, confidence interval; * Multivariable logistic regression analysis was performed using incidental PE as the dependent variable. Only variables showing clinical relevance or a univariable association (*p* < 0.10) were considered for inclusion in the multivariable model. Given the limited number of incidental PE events (n = 8), model complexity was intentionally restricted to avoid overfitting.

**Table 6:** Sensitivity analysis restricted to symptomatic pulmonary embolism: comparison between symptomatic cancer-associated pulmonary embolism (CAPE) and symptomatic non-cancer pulmonary embolism (NCPE).

Variable	Symptomatic CAPE (n = 50)	Symptomatic NCPE (n = 317)	Effect Size (95% CI)	*p*-Value
Baseline characteristics				
Age, years	71.5 ± 11.2	70.2 ± 13.4	-	0.482
Male sex, n (%)	24 (48.0%)	170 (53.6%)		0.478
Hemoglobin, g/dL	11.6 ± 1.9	13.2 ± 1.8	MD 1.60 (1.07 to 2.13)	<0.001
PESI score	131.8 ± 28.7	110.1 ± 32.3	MD 21.7 (12.9 to 30.5)	<0.001
sPESI score	3.05 ± 0.55	2.68 ± 0.81	MD 0.37 (0.16 to 0.58)	<0.001
Markers of PE severity				
RVD, n (%)	15 (30.0%)	91 (28.7%)	-	0.844
Concomitant DVT, n (%)	11 (22.0%)	84 (26.5%)	-	0.500
Primary endpoint and clinical severity				
SHPCE *, n (%)	7 (14.0%)	57 (18.0%)	-	0.492
Shock, n (%)	4 (8.0%)	42 (13.2%)	-	0.346
High-risk PE	6 (12.0%)	28 (8.8%)	-	0.468
Management-related outcomes				
ICU admission, n (%)	6 (12.0%)	37 (11.7%)	-	0.947
Systemic thrombolysis, n (%)	1 (2.0%)	17 (5.4%)	-	0.337
Early clinical outcomes				
In-hospital mortality, n (%)	6 (12.0%)	36 (11.4%)	-	0.908
Recurrent VTE, n (%)	3 (6.0%)	37 (11.7%)	-	0.289
Major bleeding, n (%)	0 (0.0%)	4 (1.3%)	-	1.000

Abbreviations: CAPE, cancer-associated pulmonary embolism; CI, confidence interval; DVT, deep vein thrombosis; ICU, intensive care unit; MD, mean difference; NCPE, non-cancer pulmonary embolism; OR, odds ratio; PE, pulmonary embolism; PESI, Pulmonary Embolism Severity Index; sPESI, simplified Pulmonary Embolism Severity Index; RVD, right ventricular dysfunction; VTE, venous thromboembolism; * SHPCE, Severe Hemodynamic Presentation Composite Endpoint: occurrence of shock, systolic blood pressure < 90 mmHg, and/or high-risk PE according to ESC risk stratification. Components of SHPCE were not mutually exclusive and may overlap. Data are presented as mean ± standard deviation (SD) or number (%), as appropriate. Odds ratios (ORs) with 95% confidence intervals (CIs) are reported for categorical variables. Mean differences (MDs) with 95% CIs are reported for continuous variables; statistically significant differences are highlighted by *p*-values <0.05.

## Data Availability

The data underlying this study were collected as part of routine clinical care and analyzed retrospectively. Due to institutional and ethical restrictions related to patient confidentiality, the dataset is not publicly available. De-identified data may be made available from the corresponding author upon reasonable request and subject to institutional approval.

## Supplementary Materials

The following supporting information can be downloaded at: https://www.mdpi.com/article/10.3390/jcm15156092/s1, Table S1. Additional biological and imaging characteristics in the sensitivity analysis restricted to symptomatic pulmonary embolism.
