# Supplementary material for: Cancer-Associated Pulmonary Embolism in Contemporary Clinical Practice: Clinical Severity, Incidental Detection, and Early Outcomes in a Real-World Cohort
Source: J Clin Med. 2026 Aug 5;15(15):6092. doi: 10.3390/jcm15156092 (PMC13467394; doi:10.3390/jcm15156092)
Supplement: Supplementary file 1 [file jcm-15-06092-s001.zip › jcm-4459283-supplementary.pdf]

Supplementary materials

Table S1. Additional biological and imaging characteristics in the sensitivity analysis restricted to symptomatic pulmonary embolism.

**Variable**	**Symptomatic CAPE (n = 50)**	**Symptomatic NCPE (n = 317)**	***p*-Value**
Biological variables			
Creatinine, mg/dL	0.91 (0.72–1.19)	0.88 (0.70–1.15)	0.662
Platelet count, ×10⁹/L	208 (159–263)	196 (155–244)	0.736
D-dimer, ng/mL FEU	5.0 (5.0–5.0)	5.0 (5.0–5.0)	0.727
Troponin, ng/L	28.5 (11.5–168.0)	43.5 (11.8–156.5)	0.750
Elevated troponin (>14 ng/L), n/N (%)	31/47 (66.0)	191/266 (71.8)	0.486
NT-proBNP, pg/mL	1604 (280–5208)	2443 (475–7506)	0.335
Imaging characteristics			
Bilateral PE, n (%)	34 (68.0)	209 (65.9)	0.873
Central PE, n (%)	0 (0.0)	17 (5.4)	0.084
Lobar PE, n (%)	40 (80.0)	209 (65.9)	0.067
Segmental/subsegmental PE, n (%)	9 (18.0)	86 (27.1)	0.218
Right ventricular dysfunction, n (%)	15 (30.0)	90 (28.4)	0.867
RV/LV ratio	0.79 (0.65–0.93)	0.75 (0.65–0.87)	0.148
TAPSE, mm	17.5 (14–22)	19 (15–22)	0.893
sPAP, mmHg	40.5 (25.8–53.8)	37 (28–50)	0.671
Concomitant DVT, n (%)	11 (22.0)	83 (26.2)	0.604

Abbreviations: CAPE, cancer-associated pulmonary embolism; NCPE, non-cancer pulmonary embolism; DVT, deep vein thrombosis; FEU, fibrinogen equivalent units; NT-proBNP, N-terminal pro-B-type natriuretic peptide; PE, pulmonary embolism; RV/LV, right ventricular-to-left ventricular diameter ratio; RVD, right ventricular dysfunction; sPAP, systolic pulmonary artery pressure; TAPSE, tricuspid annular plane systolic excursion. Data are presented as median (interquartile range) or n (%), as appropriate. Percentages for elevated troponin were calculated using available cases. This supplementary analysis was performed after exclusion of all incidentally diagnosed PE cases.
